# Critical Care Providers’ Moral Distress: Frequency, Burden, and Potential Resources

**DOI:** 10.3390/ijerph20010333

**Published:** 2022-12-26

**Authors:** Dominik Hinzmann, Katharina Schütte-Nütgen, Arndt Büssing, Olaf Boenisch, Hans-Jörg Busch, Christoph Dodt, Patrick Friederich, Matthias Kochanek, Guido Michels, Eckhard Frick

**Affiliations:** 1 Department of Anaesthesiology and Intensive Care, University Hospital Rechts der Isar, 81675 Munich, Germany; 2 School of Medicine, Technical University of Munich, 80333 München, Germany; 3 Department of Palliative Care, University Hospital Freiburg, 79106 Freiburg, Germany; 4 Quality of Life, Spirituality and Coping, Faculty of Health, Witten/Herdecke University, 58455 Witten, Germany; 5 Department of Intensive Care, University Hospital Hamburg-Eppendorf, 20251 Hamburg, Germany; 6 Department of Emergency Medicine, University Hospital Freiburg, 79106 Freiburg, Germany; 7 Emergency Department, München Klinik, 81925 Munich, Germany; 8 Department of Intensive Care, München Klinik, 81925 Munich, Germany; 9 Department of Intensive Care, University Hospital Cologne, 50937 Cologne, Germany; 10 Emergency Department, Sankt Antonius Hospital, 52249 Eschweiler, Germany; 11 Spiritual Care and Psychosomatic Health, Department of Psychosomatic Medicine and Psychotherapy, University Hospital Rechts der Isar, 80539 Munich, Germany

**Keywords:** moral distress, moral suffering, emergency department, intensive care unit, spirituality

## Abstract

Background: Critical Care Providers (CCPs) experience situations that challenge their ethics and professional standards and may entail moral distress (MD). Aim: To analyze MD perceived by CCPs in intensive care units (ICUs) or emergency departments (EDs) and further clarify whether CCPs who rely on spiritual resources differ in their perception of MD from those who do not utilize these resources. Methods: A cross-sectional anonymous survey was administered using a modified version of the German language version of the Moral Distress Scale (MDS) with 2 × 12 items to assess the frequency and the respective perceived burden of specific situations by applying a 5-point Likert scale. Explorative factor analysis was performed and the sub-constructs of the respective items regarding MD frequency and burden were identified. Job burden and professional satisfaction were measured using visual analogue scales (VAS) and a four-point Likert scale, respectively. The 15-item SpREUK questionnaire was applied to measure spiritual attitudes and behaviours and to differentiate between religious and spiritual persons. Data from 385 German-speaking CCPs were included (55% physicians, 45% nurses). Results: Conflict situations are similar for physicians and nurses although they are perceived as more burdensome by nurses. Among physicians, the MDS factor Looking away/Resignation scores highest for assistant physician residents, whereas distress caused by looking away is more often perceived by specialist physicians without a managerial position. Work satisfaction is inversely associated with MD and emotional exhaustion is positively associated with it. Participants’ spirituality is marginally associated with MD. The best predictors of both MD frequency and burden are emotional exhaustion with further influences of work satisfaction, being a nurse, and being a non-believer on the frequency of MD perception. Being a nurse, participants’ experience in ICU/ED, and being of the male gender are further predictors of MD burden. Conclusions: MD is experienced differently by different groups of CCPs depending on their place in the hierarchy of responsibility. As MD perception is best predicted by emotional exhaustion, these situations should be avoided. Although some CCPs may rely on spiritual resources, all need individual and team support to cope with MD.

## 1. Introduction

Critical care providers (CCPs) frequently perceive a conflict between how they could act and how they should act, which has been aggravated but not caused by the COVID-19 pandemic. In this context, both patients and CCPs need supportive care, which should include managing staff distress and focusing on appropriate coping strategies, one of which could be spiritual care [1]. The mismatch between CCPs’ moral beliefs and moral transgressions (experienced as a victim, witness, or perpetrator) often leads to moral suffering [2]. Moral suffering has been conceptualized as moral distress, especially in nursing [3]; moral injury, especially in the military [4]; and demoralization in healthcare (originally in cancer patients [5] but now applied to helping professions), which is the deterioration of morale and teamwork.

The current study uses the term “moral distress” (MD), coined by Jameton [3] as “the experience of knowing the right thing to do while being in a situation in which it is nearly impossible to do it”, and applies it to all CCPs. CCPs’ MD and, more generally, moral suffering, increases CCPs’ burnout and existential stress. Conversely, spirituality or a person’s faith (be it religious or not) may buffer MD perception [6,7,8]. A recent scoping review on CCPs’ MD and existential, religious, and spiritual resources [9] reported 162 eligible studies between 2020 and February 2022, but of these only 13 thoroughly examined the complex construct spirituality.

Fostering resilience encompasses improving team morale (i.e., mitigating demoralization) [10]. Some recent studies [8,11,12,13,14,15,16,17,18,19] have explored the associations between resilience and MD. In particular, the associations between emotional exhaustion (a major dimension of burnout) and MD have been explored [8,10,15,20,21,22,23,24,25,26,27]. Other studies have examined the impact of job (dis)satisfaction and the intention to leave the profession, focusing on nurses [6,28,29,30,31] and physicians [32]. Furthermore, time-related variables, such as weekly working hours [16,23,33,34,35,36,37] and years of employment [31,33,38,39,40], may be related to MD.

Further, a person’s level in hospital and ward hierarchies and a team’s ethical climate can both contribute to MD perception [41,42,43,44]. This may depend on gender aspects [26,27,35] and a CCP’s experience in the ED/ICU [30,33,45,46].

The present paper provides the quantitative results of a mixed-method study examining CCPs’ MD and their available resources. It addresses the following research questions:

1. Which situations were perceived as particularly burdensome?
2. Do nurses and physicians differ in their perceptions of MD?
3. What are the predictors of MD frequency and burden?
4. Does spirituality mitigate or enhance CCPs’ MD?

## 2. Methods

### 2.1. Description of Participants

Participants were anonymously recruited between January and March 2022 by snowball sampling, a method of convenience sampling using a chain-referral technique, which collected data through existing structures, e.g., professional associations of CCPs. An online survey link was sent to the group members. The anonym survey comprised the study information and an internet-based informed consent form, providing information about the description of the study, participants’ rights, the researcher’s contact details, and the eligibility criteria. To indicate informed consent, participants had to click the “consent” box. No incentives were offered to the participants.

### 2.2. Measures

#### 2.2.1. Moral Distress

To measure health care professionals’ MD, we used a modified version (version 2) of the German language version of the Moral Distress Scale (MDS) with 2 × 12 items [47]. The instrument uses 12 items to assess the frequency of experiences in specific situations and 12 items to assess the respective perceived burden. Using Rasch analysis, the authors improved the primary scales of Hamric and Blackhall’s version of Corley’s MDS [48]. However, the authors did not provide internal consistency coefficients.

As Kleinknecht-Dolf et al. [47] recommended the usage of sum scores that would obscure the underlying factors, we performed an explorative factor analysis [49].

Participants had to indicate the frequency of perception on a 5-point Likert scale (0 = never–4 = very often), whereas the perceived disturbance or burden because of these experiences was rated on a second 5-point Likert scale (0 = none to 4 = very high).

#### 2.2.2. Job Burden

Perception of burden was assessed with two visual analogue scales (VAS) ranging from 0 (not at all) to 100 (extremely). One VAS asked about participants’ current feelings of being burdened and the other about feeling emotionally exhausted because of their job.

#### 2.2.3. Professional Satisfaction

Participants’ job satisfaction was rated on a 4-point Likert scale ranging from very dissatisfied (0) to very satisfied (4).

#### 2.2.4. Spiritual Attitudes and Behaviours

To assess whether people were in search of a spiritual source to cope with difficult situations, whether they had already found such a resource, and how the experience of difficult situations may have changed their behaviour, we used the 15-item SpREUK questionnaire [50,51]. It avoids explicit religious terminology and is thus also suitable for non-religious people. The instrument differentiates 3 factors:

(1) *
Search* (*for Support/Access*): Cronbach’s alpha = 0.91; 5 items, e.g., F1.5—My situation has led me to deal intensively with spiritual or religious questions again; F1.6—I am convinced that my situation can be positively influenced if I can find access to a spiritual source; F1.9—I am looking for access to spirituality/religiousness.
(2) *Trust* (*in Higher Guidance/Source*): Cronbach’s alpha = 0.91; 5 items, e.g., F37—Due to my (stressful) life situation, I get to think about what is really important to me in my life; F38—I have faith in spiritual guidance in my life; F39—I feel connected to a “higher source”.
(3) *Reflection* (*Positive Interpretation of Disease*): Cronbach’s alpha = 0.86; 5 items, e.g., F3.3—What happens to me is a clue to change something in my life; F3.4—Due to my (stressful) situation, I am able to deal more with myself again; F3.7—I see my (stressful) life situation as an opportunity for my personal development.

All items were rated on a 5-point scale from disagreement to agreement. The scores can be referred to as a 100% level (transformed scale score). Scores > 60% indicate a positive attitude, scores between 40 and 60 indicate indifference, and scores < 40 indicate a negative attitude.

The responses for two differentiating items (religious person [R] vs. spiritual person [S]) in the SpREUK questionnaire were used to categorize the participants (R+S+), (R+S−), (R−S+), or (R−S−).

The frequency of religious/spiritual activity, e.g., praying and/or meditation, was assessed with a combined 4-fold item (0–3).

#### 2.2.5. Statistical Analyses

Descriptive statistics are presented as frequencies for categorical variables (%) and as means (±standard deviation, SD) for numerical variables. The between-group comparisons for the categorical variables were performed using Pearson’s χ^2^ Independence Test. Analysis of variance (ANOVA) and linear regression analysis were computed with SPSS 28.0. Given the exploratory nature of this study, we set a stricter significance level at *p* < 0.001.

## 3. Results

### 3.1. Description of Participants

The survey was accessed by 686 people but not all of them gave consent to participate or provided basic information. After eliminating these, 555 people remained. Of these, 385 people provided largely complete information (69% “completers”), 106 people provided some basic information but did not answer the questions about MD (19% “partial completers”), whereas 64 provided too little differentiating information and were therefore not taken into account (12%). There were no significant differences in relation to gender, mean age, or profession for these three groups (not shown). In the following sub-sections, the focus is on the dataset of the completers (n = 385).

The genders and professions of the participants were fairly well balanced, with women (54%) and physicians being more common (55%). For the latter, head physicians and senior physicians, in particular, were more common (Table S1). Job satisfaction was in the upper satisfaction range, but feelings of stress and emotional exhaustion were clearly present (Table S1).

### 3.2. Moral Distress Scale

It was found that the 12-item frequency scale (Cronbach’s alpha = 0.84) had three sub-constructs, which were Frequency: External causes (six items, Cronbach’s alpha = 0.80: h1, h6, h8, h9, h10, h12); Frequency: Looking away/resignation (four items, Cronbach’s alpha = 0.59: h4, b5, h7, h11); and Frequency: Inappropriate acting (two items, Cronbach’s alpha = 0.64: h2, h3). The burden scale (Cronbach’s alpha = 0.89) had two sub-constructs which were MDS burden: External causes (nine items, Cronbach’s alpha = 0.88: b1, b2, b3, b6, b8, b9, b10, b11, b12) and MDS burden: Looking away (three items, Cronbach’s alpha = 0.76: b4, h5, b7). Thus, two factors of the frequency scale had less convincing internal consistency, whereas the other scales had better quality. We report both the 12-item sum scores and the sum scores of the respective sub-constructs.

### 3.3. Perceived Stress in Specific Situations

Particularly stressful situations were similar for nurses (Table 1) and doctors (Table 2) (e.g., insufficient shift staffing, insufficient competence of employees [nurses vs. assistants]), lack of continuity (fluctuations, level of illness with regard to chronic illnesses), company or financial pressures). Nurses had a significantly higher sensitivity to MD than doctors.

### 3.4. MD and Perceived Stress in the Study Group

With regard to the frequency of experiencing MD and the perceived burden of MD, there were significant differences between the occupational groups (doctors vs. nurses), especially for stressful situations that were externalized (Table S2). Many of these feelings also showed gender-associated differences, with women feeling more strongly than men.

Among physicians, this feeling differed among the training groups. We found significant differences for Frequency: Looking away/resignation, which was felt more frequently by residents, and for the stress caused by looking away, which was perceived as more stressful by specialists without a managerial position (Table S2).

Job satisfaction was significantly higher among doctors than nurses, who, however, showed greater emotional exhaustion (Table S2). Job satisfaction was higher for men and emotional exhaustion was higher for women. Since these differences were not found in the differentiated group of doctors, it can be assumed that the professional groups and their different work situations had an effect.

With regard to spiritual-religious self-assessment, there were only a few trends, but there was a significant difference in job satisfaction, which was lower for R−S− people than for those with an S+R+ orientation (Table S2).

### 3.5. Correlations between MD, Stress, and Work-Associated Indicators

The indicators of MD in terms of frequency and burden were all moderately to strongly correlated with each other (Table 3). Age and professional experience in ED/ICU were weakly associated with the frequency of looking away/resignation; all other time-associated variables showed either no significant or only marginal associations with MD. However, job satisfaction was inversely associated with MD and emotional exhaustion was positively associated with it, particularly in terms of the frequency of experiencing it. The current feeling of stress at work was weakly associated with MD (Table 3). There were hardly any relevant associations for indicators of spirituality.

### 3.6. Predictors of MD Perceptions

As shown in Table 4, the best predictor of the frequency of MD perception was emotional exhaustion, followed by job dissatisfaction. These two variables variables alone explained 16% of the variance. Other significant influencing variables were the professional group, not being a religious person and, inversely, the care level of the hospital (university hospitals and maximum care providers versus hospitals providing specialist, standard, and basic care). Overall, 22% of the variance can be explained by these five variables. In the model, gender, years of employment, professional experience in ED/ICU, weekly working hours, perception of stress, religious trust, and SpR self-assessment did not have a significant influence.

The perceived MD burden is best explained by emotional exhaustion (12% explanation of variance), followed by being a nurse, little work experience in ED/ICU, and the female gender. However, only 20% of the variance can be explained overall so other influencing variables remain undiscovered. These do not include years of employment, job satisfaction, perceived stress, weekly working hours, being a religious person, or the level of care of the hospital.

## 4. Discussion

**Figure 1:**
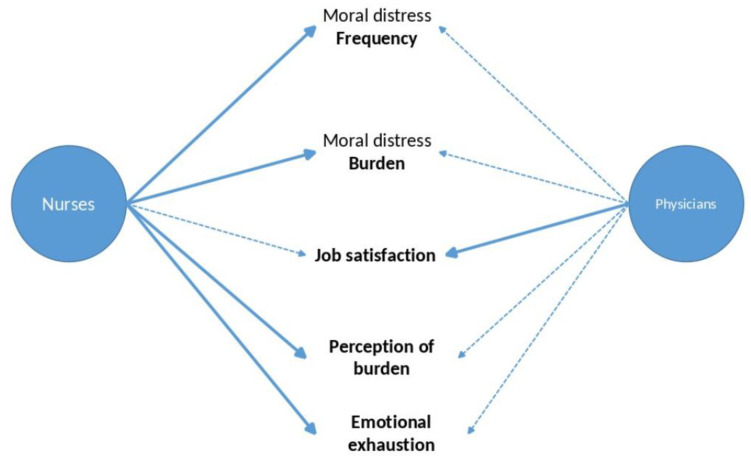
Physicians and nurses: main influences.

This cross-sectional survey investigates German-speaking CCPs’ MD and resources for the first time. Regarding the research questions formulated in the introduction of this study, we can state that (see Figure 1):

1. Both physicians and nurses perceived similar situations as burdensome, e.g., insufficient shift staffing, insufficient competence of employees, lack of continuity, and organizational or financial pressures.
2. The frequency of conflicting clinical situations provoking MD was similar for physicians and nurses, although more burdensome for nurses.
3. The best predictor of both the frequency and burden of MD was emotional exhaustion, with further influences of work satisfaction and being a nurse for the frequency of MD perception. Being a nurse, experience in ICU/ED, and being of male gender were further predictors of the burden caused by MD.
4. Being a non-believer predicted the frequency of MD perception. Participants’ underlying spirituality (in terms of the search for spirituality, religious trust, frequency of meditation or praying, and self-assessment of being a believer) was only marginally associated with MD.

### 4.1. MD in Different Professional Groups: Frequency, Burden, and Components [52]

As nurses and physicians have different roles, it is of high relevance to assess these two groups differentially. Our results confirmed previous studies maintaining that nurses were more susceptible to MD than physicians [42,53,54,55]. This could be due to their significantly closer work with the patient and the closer exchange with relatives. Nurses often see themselves as advocates for the patient in interprofessional work on the ward. These relational challenges may worsen nurses’ MD. Moreover, in the end, the physician bears the responsibility for a patient’s treatment, which might constitute a different kind of burden and stress. With different hierarchical positions and experiences of the respective professions, the impact of external and internal stressors can also vary, as well as their coping strategies [52].

Consequently, there may be a discrepancy between the apparent desire to decide on a goal for the patient, although medical parameters and laws prevent this decision path, and the handling of MD on the part of nurses. Rodríguez-Ruiz et al. [36,56,57] found that pre-pandemic, physicians suffered from significantly higher levels of MD than nurses. However, neither this comprehensive survey of Spanish CCPs nor a recent Czech study [58] constated inter-professional differences in MD during the pandemic. Conversely, post-COVID-19, physicians seem to suffer from higher burnout than nurses [37]. This is in sharp contrast to our findings, whereas Rodríguez-Ruiz et al. further reported higher MD in those who intended to leave their positions, which fits with our predictors of emotional exhaustion. Importantly, CCPs with children seemed—despite the preoccupation with their families during the pandemic—less affected by MD than CCPs without offspring [36,56,57]. Donkers et al. [59] found no differences in MD between the two groups but, interestingly, less MD compared to the pre-pandemic time. Another pre-COVID-19 study [60] observed similar MD scores in all professional groups despite the highest degree of burnout being found in nurses.

One of the MDI factors in our sample, Looking away/resignation, was similar to moral disengagement [61]. Although the MD factors External causes and Inadequate behaviour did not differ between the two professional groups [58], the factor of Looking away/Resignation was more frequent among non-specialized physicians. In addition, specialized physicians without leadership responsibilities reported a more important burden by Looking away/Resignation than those with a managerial role. However, most burdensome situations for both nurses and physicians involved the shortcomings of the staff (“insufficiently competent”, “non-partnership collaboration”), lack of continuity in the treatment team, and operational and professional pressures [58].

Our results confirm that the MD burden among physicians was associated with low levels of training and hierarchy [44,62]. It is known that MD already exists in medical students and young medical professionals who are starting their work in healthcare facilities, whereas some of them do not yet have sufficient coping skills to adequately deal with these challenges [63]. Rodríguez-Ruiz et al. [57] concluded that an age < 35 years or >50 years, without offspring, and working in the public health system were associated with higher levels of MD. In this context, special programs for medical students and young physicians that aim to convey ethical skills, in particular, should be implemented.

### 4.2. MD Predictors and Practical Consequences

In our study, the best predictors of both the frequency and burden of MD were emotional exhaustion, with further influences from the frequency of work satisfaction and being a nurse for the frequency of MD perception, whereas being a nurse, experience in ICU/ED, and being of the male gender were further predictors of the burden caused by MD. Being a nurse as a predictor of MD also underlined the assumption that a lower hierarchical position [41,42,43,44] and fewer chances to influence situations or decisions, such as therapeutic obstinacy and the provision of futile care [60] on the one hand, while working very closely with the patient on the other hand, could be a leading cause of MD. Therefore, the regular implementation of interprofessional and interdisciplinary case discussions including nurses, doctors, and other members of the team who are involved in the patient’s treatment is of particular importance, especially when ethical issues arise during the complex treatment of seriously ill patients, e.g., in therapeutic goals or the perception of futility. Regarding the relationship between MD frequency and MD burden, the so-called moral residue and being repeatedly exposed to morally distressing events have to be considered a “crescendo effect” [64]. Thus, it is necessary that interventions take place shortly after a person or team perceives a morally distressing situation.

Several studies have shown that spirituality and personal religious beliefs may be used as resources for coping with difficult situations in EDs or ICUs [62,65,66,67,68,69,70,71]. In our results, however, CCPs’ spirituality was only marginally related to their MD. Conversely, in the regression model, being a believer was a weak negative predictor of MD frequency. This means that a believing CCP would experience (or report) less MD and vice versa. Furthermore, CCPs’ self-declaration as being both religious and spiritual (R+S+) was associated with higher professional satisfaction compared with being non-religious or non-spiritual. This may indicate that their spirituality can, to some extent, be a resource [12,72,73] but may not buffer the way they perceive the conflicting situation. Nevertheless, our data do not permit us to draw far-reaching practical conclusions as far as CCPs’ spirituality is concerned.

Wang et al. [74] found that Chinese CCPs’ spirituality was positively (not negatively) related to moral injury. Spiritual CCPs may thus be more prone to MD than non-spiritual CCPs. This would indicate that a person’s spirituality may sensitize them to negative job aspects and internal perceptions such as feelings of guilt towards God. Less spiritual CCPs, conversely, perceive ethically demanding situations to be caused by external factors. Moreover, the sensitivity to MD among CCPs can vary depending on their respective moral concepts and values, which might also be related to a certain spiritual background [75,76]. CCPs experiencing MD often have limited ability to access resources, among them spiritual resources [68,77,78,79].

All in all, it has to be noted that MD in the healthcare system can never be completely avoided as CCPs will always face situations in which they have to deal with ethically challenging decisions and prioritisation issues. However, spirituality can create a “safe and scared space” [66] where CCPs can rest and energize without being judged [66,67]. Interventions co-facilitated by mental health and spiritual care providers [80] may be a new way to address shame and avoidance associated with moral suffering.

## 5. Limitations

This study relies on cross-sectional data, and thus the causality of effects is unclear. Participants were recruited in various hospitals in German-speaking countries, mostly University hospitals and maximum care hospitals, whereas primary care hospitals were underrepresented. As in universities and maximum care hospitals, the CCPs might experience medical futility more often, and this could have an impact on the burden and frequency of MD. Due to snowball sampling, we do not assume that our data are representative of all German-speaking hospitals. Furthermore, younger assistant doctors were not adequately reached. Given the fact that work experience can influence MD and that medical students and young professionals suffer from MD, the specific aspects and predictors of MD in this group might be underrepresented in the present study. In addition, the convenience sampling may have discouraged those CCPs who do not consider spirituality important. Finally, the present paper is part of a mixed-method study. Given the limitations of quantitative studies, further qualitative data are necessary to more comprehensively analyze the MD burden of CCPs.

## 6. Conclusions

Generally speaking, MD has individual and collective systemic aspects [34]. MD is partially structural distress. The individual CCP’s suffering, his or her coping resources, and the work environment and climate should be considered together. In line with Jameton’s [3] seminal work, the present study found “internal” and “external” MD constraints. MD should not be over-individualized [81]. If the focus is only on developing coping strategies without addressing the systemic causes of MD, the risk of permanent moral injury is worsened. Spirituality, in particular, is highly personal and in continental Europe it is a privatized potential resource. Consequently, in order to make spirituality accessible for coping with MD, CCPs need professional support from their organization in order to foster team spirit, open workplace spirituality [82,83], and a rational ethical climate [42].

## 7. Key Messages

Moral distress and resilience both have individual and collective systemic aspects.

In acute and intensive care, the individuals’ suffering, their coping resources, as well as the work environment and climate should be considered together.

Critical care providers need professional support from their organization in order to improve team spirit and work climate.

## Figures and Tables

**Table 1:** Particularly stressful situations for nurses.

	MW ± SD [0–4]	Perception of Burden (%)				
		None	Little	Moderate	Large	Very Large
I worked in a nursing team with shift staffing that I considered insufficient (item b9).	2.86 ± 1.14	4.4	11.3	12.5	37.5	34.4
I have worked with people from my own or another professional group who I found to be insufficiently competent so the quality of care suffered or patient safety was endangered (item b6).	2.45 ± 1.14	4.9	16.7	27.8	29.6	21.0
I have not been able to provide care that is consistent with my professional ethics due to operational or financial pressures (item b1).	2.38 ± 1.14	5.6	19.1	23.5	35.2	16.7
I observed that the quality of care and treatment suffered due to a lack of continuity in the treatment team (item b8).	2.37 ± 0.96	3.1	13.0	39.8	32.2	11.8
I have not been able to avoid or alleviate a patient’s suffering due to inadequate prescriptions (item b11).	2.25 ± 1.33	11.6	20.0	24.5	20.0	23.9

**Table 2:** Particularly stressful situations for physicians.

	MW ± SD [0–4]	Perception of Burden (%)				
		None	Little	Moderate	Large	Very Large
I worked in a nursing team with shift staffing that I considered insufficient (item b9).	2.24 ± 1.18	7.1	22.5	25.3	29.1	15.8
I observed that the quality of care and treatment suffered due to a lack of continuity in the treatment team (item b8).	2.07 ± 1.05	5.8	25.3	34.7	24.7	9.5
I have worked with people from my own or another professional group who I found to be insufficiently competent so the quality of care suffered or patient safety was endangered (item b6).	2.05 ± 1.05	6.9	25.4	31.7	27.5	8.5
I have not been able to provide care that is consistent with my professional ethics due to operational or financial pressures (item b1).	1.76 ± 1.25	17.9	27.4	26.8	16.8	11.1
I have not been able to provide care that is consistent with my professional ethics due to non-partnership collaboration (item b10).	1.70 ± 1.21	18.0	30.3	24.2	19.1	8.4

**Table 3:** Correlations between moral distress, stress and work-associated indicators.

	MDI Frequency	MDI Frequency: External Causes	MDI Frequency: Looking Away/Resignation	MDI Frequency: Inappropriate Acting	MDI Burden	MDI Burden: External Causes	MDI Burden: Looking Away
MDI Frequency	1.000						
MDI Frequency: External causes	0.932 **	1.000					
MDI Frequency: Looking away/resignation	0.693 **	0.517 **	1.000				
MDI Frequency: Inappropriate acting	0.656 **	0.457 **	0.377 **	1.000			
MDI Burden	0.761 **	0.670 **	0.749 **	0.474 **	1.000		
MDI Burden: External causes	0.765 **	0.701 **	0.629 **	0.508 **	0.967 **	1.000	
MDI Burden: Looking away	0.545 **	0.406 **	0.762 **	0.247 **	0.668 **	0.499 **	1.000
Age (years)	−0.167 **	−0.126	−0.205 **	−0.097	−0.204 **	−0.187 **	−0.180 **
Employment (years)	−0.096	−0.062	−0.157 **	−0.042	−0.108	−0.080	−0.157 **
Experience in emergency/intensive care medicine	−0.081	−0.038	−0.207 **	−0.003	−0.161 **	−0.120	−0.194 **
Working hours per week	−0.156 **	−0.126	−0.164 **	−0.104	−0.183 **	−0.196 **	−0.109
Professional satisfaction	−0.335 **	−0.328 **	−0.226 **	−0.224 **	−0.285 **	−0.308 **	−0.157 **
Perception of burden/stress	0.264 **	0.250 **	0.182 **	0.199 **	0.252 **	0.271 **	0.110
Emotional exhaustion	0.315 **	0.274 **	0.264 **	0.255**	0.342 **	0.359 **	0.161 **
SpREUK: Search	0.012	−0.019	0.105	−0.030	0.075	0.043	0.142 **
SpREUK: Trust	−0.140 **	−0.153 **	−0.028	−0.110	−0.053	−0.082	0.042
SpREUK: Reflection	0.061	0.040	0.095	0.053	0.118	0.092	0.115
Self-perception of being a believer	−0.135 **	−0.137 **	−0.039	−0.130	−0.062	−0.083	0.015
Frequency of prayer/meditation	−0.037	−0.045	0.022	−0.026	0.004	−0.018	0.043

** *p* < 0.001 (Spearman rho); moderate and strong associations were highlighted (bold).

**Table 4:** Predictors of perceived moral distress (stepwise regressions).

Dependent Variable: MDI Frequency Model 5: * F = 17.9. *p* < 0.001; R2 = 0.22	Unstandardized Regression Coefficient B	Standard Error	Standardized Beta	T	*p*
(constant)	19.997	2.694		7.424	<0.0001
Job satisfaction	−1.456	0.502	−0.167	−2.898	0.004
Emotional Exhaustion	0.076	0.018	0.239	4.225	<0.0001
Profession: Nurses	2.434	0.809	0.155	3.007	0.003
Believer	−1.014	0.370	−0.137	−2.737	0.007
Level of care of the hospital	−1.145	0.451	−0.127	−2.536	0.012
Dependent variable: MDI Burden Model 3: ** F = 19.8. *p* < 0.001; R2 = 0.20	Unstandardized Regression Coefficient B	Standard error	Standardized Beta	T	*p*
(constant)	13.498	2.758		4.895	<0.0001
Emotional Exhaustion	0.115	0.019	0.303	5.943	<0.0001
Profession: Nurses	3.718	0.995	0.200	3.737	<0.0001
Experience in emergency/intensive care medicine	−0.630	0.296	−0.109	−2.129	0.034
Female gender	−2.022	1.012	−0.108	−1.998	0.047

* Without significant influence in the model: gender, years of employment, professional experience in emergency/intensive care medicine, weekly working hours, perception of stress, religious trust. ** Without significant influence in the model: years of employment, job satisfaction, perception of stress, weekly working time, believer, care level of the hospital.

## Data Availability

The datasets used and/or analysed during the current study are available from the corresponding author upon reasonable request.

## Supplementary Materials

The following supporting information can be downloaded at: https://www.mdpi.com/article/10.3390/ijerph20010333/s1, Table S1: Descriptions of participants (n = 385); Table S2: Moral distress and perceived burden in the study group.

## Abbreviations

CCP	Critical care provider
ED	Emergency Department
ICU	Intensive Care Unit
MD	Moral Distress
MDI	Moral Distress Intensity
MDS	Moral Distress Scale
R	Religion
Sp	Spirituality
