# Supplementary material for: Critical Care Providers’ Moral Distress: Frequency, Burden, and Potential Resources
Source: Int J Environ Res Public Health. 2022 Dec 26;20(1):333. doi: 10.3390/ijerph20010333 (PMC9819312; doi:10.3390/ijerph20010333)
Supplement: Supplementary file 1 [file ijerph-20-00333-s001.zip › Supplemental Table S1.pdf]

Supplementary S1. This supplementary material was part of the submitted manuscript and has been peer reviewed. It is posted as supplied by the authors

Table S1: Descriptions of participants (n=385)

	N	% *	MW ± SD [range]
Gender<br>women<br>men<br>divers	385<br>206<br>179<br>0	100<br>53.5<br>46.5<br>0	
Mean age (years)	380		43.6 ± 10.9 [21-69]
Partner status<br>Living with partner<br>Living alone (incl. divorced and widowed)	384<br>304<br>80	100<br>79.2<br>20.8	
Religious denomination<br>Catholic<br>Protestant<br>other<br>none	384<br>152<br>119<br>18<br>95	100<br>39.6<br>31.0<br>4.7<br>24.7	
Spiritual-religious self-assessment<br>R+S+<br>R+S-<br>R-S+<br>R-S-	366<br>84<br>58<br>49<br>175	100<br>23.0<br>15.8<br>13.4<br>47.8	
Spiritual-religious self-assessment<br>R+S+/R+S-/R-S+<br>R-S-	366<br>191<br>175	100<br>52.2<br>47.8	
Regarded as religious person<br>Not at all<br>Rather no<br>Yes, somewhat<br>Yes, definitely	385<br>76<br>96<br>123<br>90	1100<br>19.7<br>24.9<br>31.9<br>23.4	
Prayer / meditation frequency<br>not at all<br>rather seldom<br>sometimes<br>regularly<br>Profession<br>Chief / Head doctors<br>Senior physician<br>Specialist Physician (without management position)<br>Assistant physician<br>Nurse<br>other	554<br>202<br>129<br>129<br>94<br>379<br>40<br>100<br>40<br>25<br>167<br>7	100<br>36.5<br>22.3<br>22.3<br>17.9<br>100<br>10.6<br>26.4<br>10.6<br>6.6<br>44.1<br>1.8	1.21 ± 1.11 [0-3]
Profession<br>Medical doctors<br>Nurses	376<br>205<br>167	 55.1<br>44.9	
Additional/specialist training for doctors **<br>Intensive care<br>Acute/emergency medicine<br>Emergency medicine<br>Palliative medicine	 113<br>49<br>121<br>20	 55.1<br>23.9<br>59.0<br>9.8	

Additional/specialist training for nurses **<br>Emergency care<br>Intensive care<br>Palliative care	 16<br>101<br>14	 9.6<br>60.5<br>8.4	
Specialist assignment of the intensive care unit **<br>Internal<br>Anesthesia<br>Anesthesia + Internal<br>Surgery<br>Anesthesia + Surgery<br>Neurology / Neurosurgery<br>Pediatrics / Neonatology<br>other	 130<br>64<br>126<br>29<br>76<br>64<br>11<br>35	 33.8<br>16.6<br>32.7<br>7.5<br>19.7<br>16.6<br>2.9<br>9.1	
Specialist assignment of the emergency room<br>Conservative<br>Surgical<br>Interdisciplinary<br>Pediatric	371<br>51<br>24<br>284<br>12	100<br>13.7<br>6.5<br>76.5<br>3.2	
Level of care of the hospital<br>University hospital<br>Maximum care clinic<br>Specialty Care Clinic<br>Standard care clinic<br>Primary care clinic<br>other	381<br>126<br>98<br>74<br>51<br>20<br>12	100<br>33.1<br>25.7<br>19.4<br>13.4<br>5.2<br>3.1	
Employment (years)	377		19.2 ± 10.7 [0.5-47]
Experience in emergency/intensive care medicine	383		4.1 ± 1.6 [0-6]
Working hours per week	380		42.3 ± 13.3 [8-100]
Professional satisfaction<br>Very dissatisfied<br>Dissatisfied<br>It's going ok<br>Satisfied<br>Very satisfied	385<br>6<br>30<br>108<br>183<br>58	100<br>1.6<br>7.8<br>28.1<br>47.5<br>15.1	2.7 ± 0.9 [0-4]
Perception of Burden / Stress	381		64.5 ± 20.3 [0-100]
Emotional Exhaustion	370		58.5 ± 25.2 [0-100]

* referring to the respondents; ** multiple answers were allowed
