# Supplementary material for: Critical Care Providers’ Moral Distress: Frequency, Burden, and Potential Resources
Source: Int J Environ Res Public Health. 2022 Dec 26;20(1):333. doi: 10.3390/ijerph20010333 (PMC9819312; doi:10.3390/ijerph20010333)
Supplement: Supplementary file 1 [file ijerph-20-00333-s001.zip › Supplemental Table S2.pdf]

Table S2: Moral distress and perceived burden in the study group

		**MDI Frequency**	MDI Frequency: External causes	MDI Frequency: Looking away / resignation	MDI Frequency: Inappropriate acting	**MDI Burden**	MDI Burden: External causes	MDI Burden: Looking away	**Job satisfaction**	**Perception of burden**	**Emotional exhaustion**
All participants (n=385)	MW	19.75	12.96	3.42	3.80	19.93	17.08	2.36	2.67	64.49	58.49
	SD	8.02	5.30	2.62	1.97	9.58	7.73	2.48	0.88	20.30	25.23
range		0-48	0-24	0-16	0-12	0-48	0-36	0-12	0-4	0-100	0-100
**Profession**											
Physicians (n=205)	MW	17.82	11.90	2.83	3.48	17.30	14.86	1.98	2.85	62.84	55.18
	SD	7.90	5.33	2.39	1.94	8.93	7.25	2.24	0.80	21.03	25.27
Nurses (167)	MW	22.24	14.43	4.02	4.28	22.93	19.79	2.64	2.41	66.89	63.46
	SD	7.22	4.78	2.62	1.90	9.07	7.23	2.52	0.91	18.91	24.15
p value (t-Test)		**<0.0001**	**<0.0001**	**<0.0001**	**<0.0001**	**<0.0001**	**<0.0001**	**0.008**	**<0.0001**	n.s.	**0.002**
Cohen´s d		**0.58**	**0.50**	0.48	0.42	**0.63**	**0.68**	0.28	**0.53**	0.20	0.33
**Physician groups**											
Chief / Head doctors (n=40)	MW	15.53	10.93	1.98	2.88	14.35	12.76	1.23	3.12	64.74	50.77
	SD	7.82	5.32	2.26	1.80	9.05	7.73	1.91	0.97	24.24	26.79
Senior physicians (n=100)	MW	17.78	12.03	2.65	3.45	16.61	14.45	1.75	2.85	64.08	56.88
	SD	7.72	5.39	2.26	1.96	8.94	7.29	2.05	0.73	19.73	25.92
Specialist Physician (without management position) (n=40)	MW	18.98	12.28	3.51	3.85	20.68	16.95	3.05	2.70	58.50	51.54
	SD	8.53	5.84	2.49	1.98	7.91	5.92	2.67	0.85	21.31	24.01
	MW	19.84	12.36	3.88	3.96	19.12	16.32	2.44	2.68	62.00	61.67

Assistant physician (n=256)	SD	7.19	4.29	2.47	1.88	8.72	7.55	2.12	0.63	20.62	21.20
F value		1.99	0.59	4.81	2.34	3.86	2.59	5.57	2.45	0.80	1.34
p value (ANOVA)		n.s.	n.s.	**0.003**	n.s.	**0.010**	n.s.	**0.001**	n.s.	n.s.	n.s.
**Gender**											
women (n=206)	MW	21.20	13.76	3.92	4.07	22.30	19.07	2.61	2.55	66.18	61.96
	SD	7.80	5.06	2.67	1.96	9.34	7.48	2.54	0.90	20.59	25.06
men (n=179)	MW	18.07	12.04	2.84	3.49	17.24	14.82	2.07	2.80	62.54	54.44
	SD	7.97	5.44	2.43	1.95	9.15	7.39	2.38	0.84	19.85	24.90
p value (t-Test)		**<0.0001**	**0.001**	**<0.0001**	**0.004**	**<0.0001**	**<0.0001**	0.031	**0.004**	n.s.	**0.004**
Cohen´s d		0.40	0.33	0.42	0.30	**0.55**	**0.57**	0.22	0.29	0.18	0.30
**SpR self-assessment**											
R+S+/R+S-/R-S+ (n=191)	MW	19.25	12.58	3.45	3.63	19.56	16.63	2.49	2.80	63.89	59.61
	SD	8.27	5.30	2.79	1.98	10.17	8.13	2.62	0.82	21.30	24.82
R-S- (n=175)	MW	20.79	13.77	3.46	4.04	20.78	17.96	2.30	2.51	65.32	58.06
	SD	7.51	5.10	2.45	1.94	8.83	7.15	2.37	0.95	19.37	25.50
p value (t-Test)		n.s.	0.030	n.s.	0.045	n.s.	n.s.	n.s.	**0.002**	n.s.	n.s.
Cohen´s d		0.20	0.23	0.00	0.21	0.13	0.17	0.08	0.33	0.07	0.06
**Level of care of the hospital**											
University hospital (n=126)	MW	21.41	13.90	3.77	4.21	20.90	18.00	2.46	2.60	64.27	58.33
	SD	7.95	5.29	2.39	1.94	9.11	7.55	2.32	0.84	21.15	25.35
	MW	19.74	13.14	3.29	3.76	20.15	17.16	2.45	2.63	65.51	58.42

Maximum care clinic (n=98)	SD	8.56	5.70	2.75	2.14	10.10	8.06	2.62	0.96	19.85	24.73
Speciality, Standard and Primary care clinics (n=157)	MW	18.33	12.05	3.17	3.50	18.77	16.15	2.13	2.75	64.39	59.01
	SD	7.49	4.94	2.67	1.83	9.56	7.66	2.41	0.87	19.91	25.68
F value		5.28	4.43	1.94	**4.57**	1.78	1.99	0.86	1.11	0.12	0.03
p value (ANOVA)		**0.005**	**0.012**	n.s.	**0.011**	n.s.	n.s.	n.s.	n.s.	n.s.	n.s.

Moderate effect sizes (Cohen´s d > 0.5) are highlighted
